# Transcatheter aortic valve replacement with balloon-expandable valve: Analysis of initial experience in China

**DOI:** 10.1007/s00059-017-4622-x

**Published:** 2017-12-13

**Authors:** Y. Shen, H. Zhang, L. Zhang, H. Li, H. Mao, Y. Pei, Z. Jing, Q. Lu

**Affiliations:** Department of Vascular Surgery, Changhai Hospital, Shanghai, China

**Keywords:** Aortic valve stenosis, Coronary angiography, Minimally invasive surgery, Patient selection, China, Aortenklappenstenose, Koronarangiographie, Minimalinvasive Chirurgie, Patientenselektion, China

## Abstract

**Background:**

Transcatheter aortic valve replacement (TAVR) is widely applied for the treatment of severe aortic stenosis (AS) in developed countries; however, in China, it is still in the early stage of utilization. On the basis of previous studies, this work explored the feasibility of TAVR in patients with severe AS in China and analyzed the cause of death in four cases.

**Methods:**

This retrospective study included 20 patients who had severe AS and underwent TAVR with a balloon-expandable system (Edwards SAPIEN XT) in our hospital from January 2011 to June 2016. The valve and heart functions of 16 survivors before and after the TAVR procedure were compared. TAVR endpoints, device success, and adverse events were assessed according to the definitions of the Valve Academic Research Consortium-2 (VARC-2).

**Results:**

There were 13 male and seven female patients aged 65–81 years (average, 73.15) who underwent TAVR. The TAVR approach was transfemoral in 19 patients and transapical in one patient. Four patients died (two of coronary artery occlusion and two of aortic annulus rupture) during the TAVR procedure or shortly after; six patients had mild paravalvular leakage, and the rest of the patients showed a significant improvement in cardiac function. During the follow-up period (2–62 months), one patient died of lung cancer 13 months after the TAVR procedure.

**Conclusion:**

TAVR with a balloon-expandable system is safe and effective and can be used for patients with severe AS in China. It requires careful patient selection and preoperative assessment so as to reduce the 30-day postoperative mortality rate.

## Zusammenfassung

**Hintergrund:**

Der Transkatheter-Aortenklappenersatz („transcatheter aortic valve replacement“, TAVR) ist in Industrieländern zur Behandlung der schweren Aortenstenose (AS) weit verbreitet, in China jedoch ist er immer noch im Frühstadium der Anwendung. Auf der Grundlage vorhergehender Studien wurde in der vorliegenden Arbeit die Durchführbarkeit der TAVR-Operation bei Patienten mit schwerer AS in China untersucht und in 4 Fällen die Todesursache analysiert.

**Methoden:**

In die retrospektive Studie wurden 20 Patienten aufgenommen, bei denen eine schwere AS vorlag und eine TAVR-Operation mit einem ballondilatierbaren System (SAPIEN XT, Fa. Edwards, Irvine/CA, USA) zwischen Januar 2011 und Juni 2016 in der Klinik der Autoren erfolgte. Klappen- und Herzfunktion der 16 überlebenden Patienten wurden vor und nach der TAVR-Operation verglichen. TAVR-Endpunkte, erfolgreicher Geräteeinsatz und Nebenwirkungen wurden gemäß den Definitionen des Valve Academic Research Consortium-2 (VARC-2) ermittelt.

**Ergebnisse:**

Bei 13 männlichen und 7 weiblichen Patienten im Alter von 65 bis 81 Jahren (Durchschnitt: 73,15) wurde eine TAVR-Operation durchgeführt. Der Zugang für den TAVR war bei 19 Patienten transfemoral und bei einem Patienten transapikal. Es starben 4 Patienten (2 durch Koronararterienverschluss und 2 durch eine Ruptur des Aortenrings) während der TAVR-Operation oder kurz danach; 6 Patienten wiesen eine leichtgradige paravalvuläre Leckage auf, und bei den übrigen Patienten zeigte sich eine deutliche Verbesserung der Herzfunktion. Während der Nachbeobachtungsphase (2–62 Monate) starb ein Patient 13 Monate nach der TAVR-Operation an einem Bronchialkarzinom.

**Schlussfolgerung:**

Die TAVR-Operation mit einem ballondilatierbaren System ist sicher und wirksam und kann bei Patienten mit schwerer AS in China eingesetzt werden. Es bedarf einer sorgfältigen Patientenauswahl und präoperativen Untersuchung, um die postoperative 30-Tage-Mortalitätsrate zu senken.

Aortic stenosis (AS), either congenital or acquired, is a common aortic valve disease among the aged population. Congenital AS is mainly caused by bicuspid aortic valve. Acquired AS is often termed “senile” or “calcific valvular” AS, which is considered a degenerative process. The morbidity rate of AS patients older than 75 years is as high as 2.8% [1]. Although performing surgical aortic valve replacement (SAVR) under extracorporeal circulation is still the most common treatment for AS and can achieve good outcomes, it can also lead to significant patient trauma, inflammatory stress, and other potential risks. This is especially true for the frail older population with severe circulatory, respiratory, or urinary system disease, which greatly increases the surgical risk. Research shows that more than 40% of AS patients older than 75 years cannot tolerate SAVR [1]. At the beginning of this century, transcatheter aortic valve replacement (TAVR) heralded a new era of minimally invasive surgical repair for aortic valve diseases. In 2002, Cribier and colleagues accomplished the first TAVR in humans [2]. Cohort studies [3, 4] also showed that TAVR potentially had a lower procedural mortality than SAVR in all risk groups. TAVR was proved to be an effective and safe treatment and was carried out in developed countries for patients considered high risk for SAVR [5]. However, in developing countries, the technique is relatively new.

From 2011 to 2016, our team made a preliminary attempt to manage patients with valvular heart disease in accordance with the 2014 American Heart Association/American College of Cardiology (AHA/ACC) guideline [6]. During the procedure, transcatheter balloon-dilated aortic valve replacement (Edwards SAPIEN XT) was applied to 20 volunteers who had severe AS and were considered high risk (logistic EuroSCORE >15%) for SAVR [7, 8]. The initial outcomes were encouraging.

## Patients and methods

### Patients

From January 2011 to June 2016, 20 patients with severe AS were enrolled in this retrospective study. All patients had undergone strict preoperative evaluations using transthoracic echocardiography (TTE), coronary angiography (CAG), computed tomographic angiography (CTA), and lung function tests to assess the cardiac structure and function, systemic vascular conditions, and other comorbidities.

Besides those who refused open surgery, high-risk patients with severe symptomatic AS who were deemed inoperable included those with: (1) severe AS with an aortic valve area of less than 0.8 cm^2^ plus an aortic velocity no less than 4.0 m/s, or mean pressure gradient equal to or greater than 40 mm Hg with New York Heart Association (NYHA) functional class II or higher; (2) logistic EuroSCORE of 15% or higher. The exclusion criteria were: congenital bicuspid aortic valve, acute myocardial infarction, significant coronary artery disease (CAD), left ventricular ejection fraction (LVEF) <20%, aortic annulus area >650 mm^2^ or <300 mm^2^, severe aortic or mitral regurgitation, severe kidney dysfunction, and transient ischemic attack within 6 months [6].

The current study comprised 20 patients (13 male) who had congestive heart failure with NYHA class II–IV and a logistic EuroSCORE higher than 15%. The aortic annulus diameters ranged from 20 to 25 mm, as determined by TTE.

### Surgical technique

All operations were performed with the patients under general anesthesia; endotracheal intubation and mechanical ventilation were used during the procedure. Edwards SAPIEN XT Transcatheter Heart Valves (23 mm, 26 mm, and 29 mm) provided by Edwards Lifescience Corp. (Irvine, CA, USA) were used in all patients. Monitoring devices utilized for real-time supervision during the TAVR procedure included transesophageal echocardiography (TEE), three-channel electrocardiogram (ECG), continuous oxygen saturation, and hemodynamic assessment via radial artery catheter. A standard transfemoral retrograde approach was conducted as described by Kasel [9]. All procedures were performed via the transfemoral approach except for one patient with an aortic valve area of 0.22 cm^2^ in whom the procedure was assisted by the transapical approach.

### Statistical analysis

All data are summarized and displayed as mean±SD for continuous variables and numbers (%) for categorical variables. Valve function and heart function before and after TAVR were analyzed and compared by the Wilcoxon signed-rank test or the paired-samples *t* test (Table 1). All two-tailed test results with a value of *p* < 0.05 were considered statistically significant.

**Table 1:** Comparison of valve and heart function before and after TAVR

Characteristics of surviving patients (*n* = 16)	Before TAVR	After TAVR	*p*
LVEF (%)	59.31 ± 11.32	61.88 ± 8.49	0.132
Vmax (cm/s)	512.63 ± 75.71	232.69 ± 71.54	<0.001
NYHA class ≥ II, *n* (%)	16 (100)	3 (18.75)	<0.001

Values are mean ± SD or *n* (%)

*LVEF* left ventricular ejection fraction, *NYHA* New York Heart Association

## Results

A total of 20 patients (13 male) underwent TAVR procedures with the Edwards Sapien XT valve system in our hospital, of which 19 procedures were transfemoral and one was assisted by a transapical approach. The baseline characteristics of patients are presented in Table 2.

**Table 2:** Baseline patient characteristics

Age (years)	73.15 ± 4.52
Male	13 (65%)
Hypertension	14 (70%)
Diabetes	5 (25%)
CAD	5 (25%)
PAD	1 (5%)
AVA (cm^2^)	0.74 ± 0.24
EOAI	0.44 ± 0.14
LVEF (%)	58.40 ± 11.46
PGmax (mm Hg)	102.95 ± 32.54
Vmax (cm/s)	503.60 ± 90.06
Left coronary ostial height	13.61 ± 0.80
Right coronary ostial height	14.87 ± 1.22
Logistic EuroSCORE (%)	17.72 ± 3.38

Values are mean ± SD or *n* (%)

*CAD* coronary artery disease, *PAD* peripheral artery disease, *AVA* aortic valve area, *EOAI* effective orifice area index, *LVEF* left ventricular ejection fraction, *PGmax* maximum pressure gradient, *Vmax* maximum aortic velocity

Follow-up TTE was performed 1 month, 6 months, and 1 year after the TAVR procedure and annually thereafter. In our study, we mainly focused on the 30-day results. The results are displayed in Table 3 according to the Valve Academic Research Consortium-2 (VARC-2) definitions [10]. Four patients with a mean age of 70.5 years (range 68–72) died during or shortly after the procedure. Of these patients, one was female and two had a history of CAD. All of them had an NYHA class no higher than level III except one patient with level IV. Two patients died of acute myocardial infarction resulting from coronary occlusion, one of them had the left main coronary artery obscured by a native valve during the balloon-expanding procedure, and the other one suffered aortic sinus dissection caused by balloon expansion. Two patients died of pericardial tamponade caused by aortic annulus rupture. Six patients (30%) had mild paravalvular leakage. Neither life-threatening bleeding, vascular complications, operation-correlated cardiac arrhythmia, myocardial infarction, aortic valve displacement, nor severe paravalvular leakage were found. Vital signs were stable in the remaining patients.

**Table 3:** Outcomes of the 30-day follow-up study

Outcomes	Cases (*N* = 20)
All-cause mortality (%)	4 (20)
Cardiovascular mortality (%)	4 (20)
Non-cardiovascular mortality (%)	0 (0)
Coronary obstruction (%)	2 (10)
Cardiac tamponade (%)	2 (10)
Bleeding complications (%)	0 (0)
Vascular complications (%)	0 (0)
Conduction disturbances and arrhythmias (%)	0 (0)
Myocardial infarction (%)	0 (0)
Valve migration (%)	0 (0)
Acute kidney injury	0 (0)
Stroke	0 (0)
Paravalvular leakage	–
Mild (%)	6 (30)
Moderate (%)	0 (0)
Severe (%)	0 (0)

Values are *n* (%)

TTE showed significant improvement of cardiac function in the 30-day follow-up. The mean maximum aortic velocity decreased from 512.63 ± 75.71 cm/s to 232.69 ± 71.54 cm/s (*p* < 0.001). Moreover, 13 surviving patients showed an improvement in NYHA functional class to class I within 30 days. During the follow-up period, one patient died of lung cancer after 13 months; the rest of the 15 patients were able to resume their daily activity independently.

## Discussion

AS is a significant problem that is directly correlated with advanced age [11]. Because drug therapies have a low efficiency and do not lead to high survival rates, the treatment of severe AS has primarily been SAVR. In the past decade, the emergence of TAVR has offered a less invasive approach for the treatment of patients with severe AS [12]. For these patients, TAVR shows a lower short-term mortality rate compared with SAVR [13], and thus might be a better option. A systematic review [14] showed that TAVR had a mortality rate of 8.4%, among which multiorgan failure accounted for 20.0%, followed by cardiovascular complications and stroke, accounting for 16.0% and 12.0%, respectively. These were the three leading causes of death after TAVR with the Edwards SAPIEN Valve within 30 days according to the review. In our retrospective study, we observed initial success in 16 cases, but there were still four patients who died during the procedure or shortly after. The 30-day mortality rate of the 20 patients in our study was higher than that in the literature (4/20, 20%) [13]; besides the high-risk factors present in the patients themselves (logistic EuroSCORE > 15%), further improvements in the preoperative evaluations and implantation techniques were also required.

Here, we discuss the causes of death in our four patients.

### Coronary occlusion

The etiology of coronary occlusion is often multifactorial and generally includes asymmetric expansion caused by bicuspid valve malformation, bulky calcification on the left or right coronary cusps, low take-off of the coronary ostia, large aortic valve leaflets, and shallow aortic sinus.

**Fig. 1:**
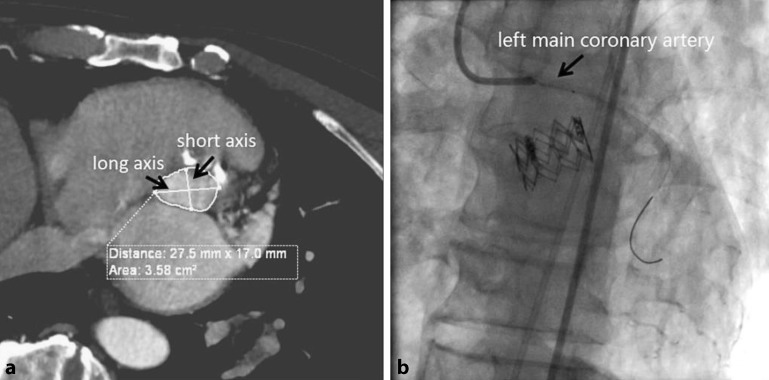
**a** Aortic annulus area and long- and short-axis diameters. **b** Coronary angiography after coronary stent implantation

Aortic sinus dissection as a rare complication caused by TAVR has not been reported in the current literature. However, it can result in myocardial infarction and was considered to be the cause of death of the first patient in our study. The preoperative CTA of the first patient showed an annulus area of 358 mm^2^ (Fig. 1a); a 23-mm artificial valve with a 17-ml balloon of 1‑ml underfill was chosen, which provided 10.0% oversizing. The take-off of the left coronary artery was 15.4 mm (lower than the right one), and was considered to be a safe distance. After releasing the valve stent, the left coronary ostium was seen to be occluded under digital subtraction angiography (DSA). After percutaneous transluminal coronary angioplasty (PTCA) and stent implantation, the blood flow of the left coronary artery recovered immediately (Fig. 1b). The patient was reported to have acute coronary syndrome (ACS) on the first postoperative day and died afterwards. The take-off of the left coronary ostia was considered safe enough and the CAG showed that the left coronary artery kept patency after stent implantation. As a result, we considered that coronary sinus dissection occurred and led to the acute occlusion of the coronary ostia. The risk factors remained unclear, but could be related to annulus calcification and the balloon-expansion procedure.

**Fig. 2:**
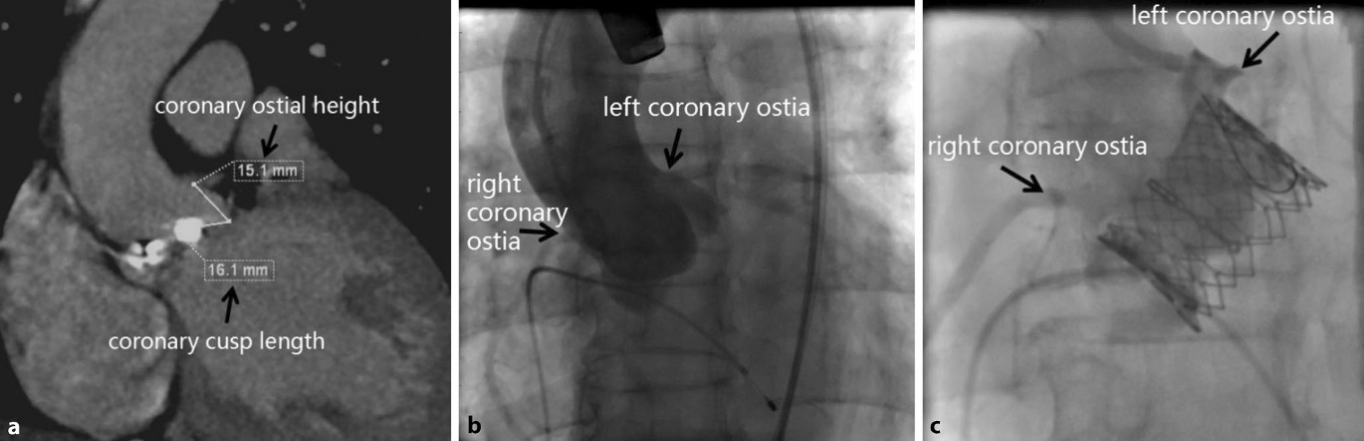
**a** Coronary ostial height and coronary cusp length. **b** Aortic root angiography before TAVR. **c** Coronary angiography indicating occlusion of the left coronary ostia

Researchers have shown that [15] a distance of <10 mm between the base of the coronary arteries and the hinge point of the corresponding cusp pose an increased risk for occlusion. Among the 20 patients, the average height of the coronary ostia was 13.64 mm (range, 12.27–15.42 mm) in the left side and 14.87 mm (range, 13.05–16.70 mm) in the right side using the three-dimensional structure software Aquarius Workstation (Version 3.7.0.13, TeraRecon, Foster City, CA, USA). In the second patient, the left coronary ostial height was 15.1 mm (lower than the right) and was considered to be a safe distance (Fig. 2a). Angiography showed that the patency of the cornary arteries were good before stent implantation (Fig. 2b). In other investigations [15], it was pointed out that if the coronary ostial height is shorter than the coronary cusp length, it could also increase the risk of coronary occlusion, which was thought to be the cause of death in the second patient. After releasing the valve stent, the left coronary ostium was covered by a self-leaflet (Fig. 2c). It indicated a shorter left main ostial height (15.1 mm) than the left coronary cusp leaflet length (16.1 mm), which was the main reason for coronary occlusion (Fig. 2a). To avoid such life-threatening situations, surgeons should analyze the preoperative imaging carefully, especially the coronary ostial height, the bulky calcification on the left or right coronary cusps, and the shape of the coronary sinus. For patients with CAD, CAG should be performed before TAVR to decide whether to implant coronary stents or not.

After stent implantation, the coronary artery blood supply and the artificial valve stent function should be examined. If there is coronary occlusion, PTCA should be performed immediately. Furthermore, a hybrid operation room should be readily available in the case of coronary artery bypass grafting (CABG).

### Aortic annulus rupture

**Fig. 3:**
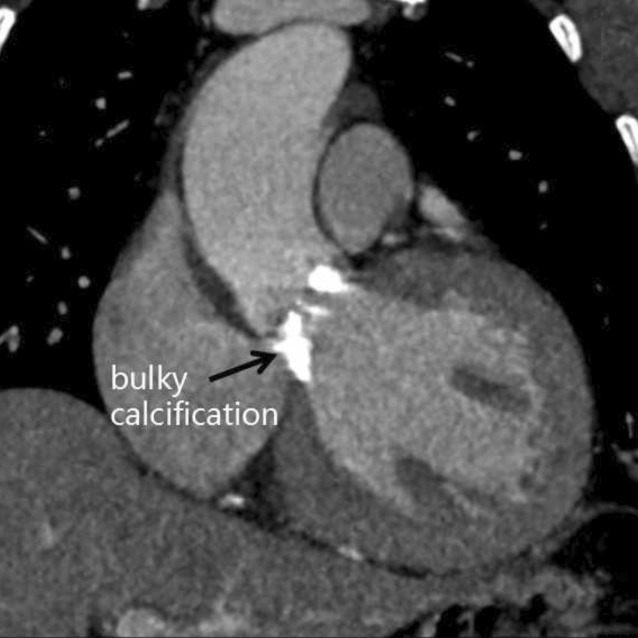
Bulky calcification in the valve deployment zone

Larger registries [16] reported that periprocedural aortic annular rupture was less than 1% of the total procedures and was more common with balloon-expandable valves. Aortic annulus rupture usually occurs because of poor patient selection, lack of experience, measurement error, aggressive dilatation, valve oversizing (>20% was shown to be associated with an 8.4-fold higher risk of contained and uncontained ruptures with the balloon-expandable system [17]), bulky calcifications in the valve deployment zone (Fig. 3; [17, 18]), severe asymmetric subvalvular hypertrophy, and global left ventricular hypertrophy in the elderly, especially in female patients with relatively weak myocardium.

**Fig. 4:**
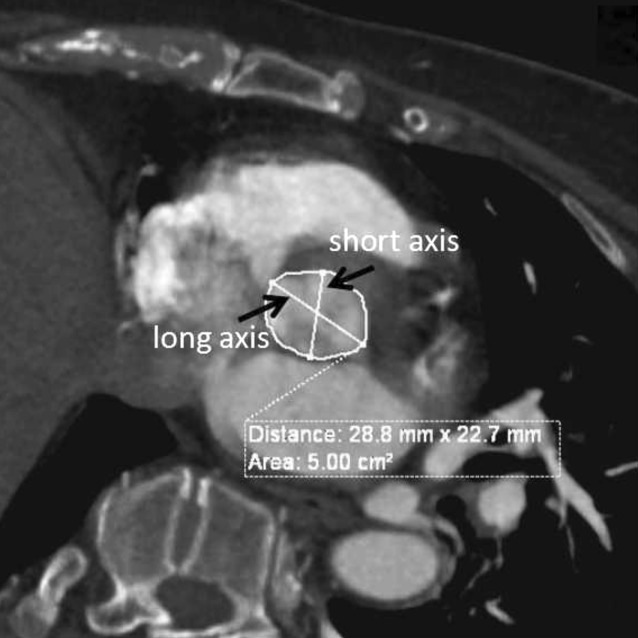
Aortic annulus area and long- and short-axis diameters

The third patient who died in our series was a 72-year-old woman with NYHA class IV. Asymmetric hypertrophy of interventricular septum and decreased left ventricular compliance were detected with echocardiography. An aortic annulus area of 500 mm^2^ with no obvious calcification was found by CTA (Fig. 4). A 26-mm valve with a 22-mm balloon was needed (6.2% oversizing). During the balloon dilatation procedure, there were temporary system errors in the pacemaker, which was observed under DSA and resulted in a slight sliding of the balloon. After balloon displacement, cardiac tamponade occurred. Pericardiocentesis was carried out and the symptoms were gradually controlled. After entering the ICU, however, the patient’s heart rate and blood pressure dropped again within 4 h and she died. Advanced age, female gender, as well as reduced cardiac structure and function were thought to be critical risk factors. The balloon displacement might be the main reason for the aortic annulus rupture.

Extensive calcification of the valve deployment zone is associated with lower device success and increases the risk of rupture during the process of balloon expansion [19]. The fourth patient in our series had an aortic annulus area of 568 mm^2^ and extensive calcification was found in the valve deployment zone via CTA. A 29-mm valve and a 23-mm balloon with 1‑ml underfill were selected, which had an oversizing of 13.3%. After balloon expansion, hydropericardium was seen under TEE observation. Although pericardiocentesis and exploratory thoracotomy were performed immediately, it still failed to save the patient’s life; the patient died of cardiac tamponade caused by aortic annulus rupture. Whether patients with extensive calcification of the valve deployment zone are suitable for a balloon-expandable valve system and how to select the rate of oversizing are issues that still need further exploration.

Selecting suitable patients and the correct size of the valve system via preoperative imaging assessment is important to reduce the rates of rupture. The rate of oversizing is recommended to be 5–20% when the Sapien XT system is used [17]. In cases of extreme calcification, an average rate of 7.5% is recommended [20] to balance the oversizing that may result in rupture and the undersizing that may result in increased paravalvular regurgitation. Balloon underfilling during valve deployment may also lower the risk of rupture. In addition, it is equally important to ensure the effectiveness and stability of the pacemaker before and during the procedure.

Acute hemodynamic collapse immediately follows the rupture, which can be observed with TEE or DSA. In order to maintain hemodynamic stability, a hybrid operating room is usually required to perform cardiopulmonary bypass and sternotomy with conventional cardiac procedures. Alternatives include isolated pericardial drainage and a conservative strategy if the rupture is contained and the patient is not in an immediate life-threatening situation. Recently, researchers described endovascular repair with the use of tissue glue injection via a microcatheter to seal an annular perforation defect [20]; this requires further validation and long-term follow-up before entering the guidelines.

### Limitations

Patient selection and assessment were highly subjective. Family members held different attitudes toward exploratory thoracotomy and autopsy, and some of them rejected the study proposal. Thus, the causes of death in some patients could only be judged by the clinical experiences. The study was a single-center, non-randomly controlled study and had a limited number of patients. It was not adequately powered for clinical or procedural endpoints with the 30-day follow-up period, and therefore further postoperative studies are required.

### Conclusion

Although several life-threatening complications occurred and four patients died during TAVR or shortly after, the procedure was successful in the other 16 patients of this study, who had significant improvements in cardiac function during the 30-day follow-up. To avoid complications, better patient selection, more accurate preoperative assessment—especially in aortic root structures—more experienced management, and timely remedial measures are necessary. For single centers with few patients and a lack of treatment experience, multi-institutional programs are needed to further establish a database of endovascular treatment in AS. With accumulation of endovascular experiences and fast-developing techniques, TAVR is expected to be widely applied in developing countries in the near future.
